# Simulated Microgravity Induces the Proliferative Inhibition and Morphological Changes in Porcine Granulosa Cells

**DOI:** 10.3390/cimb43030155

**Published:** 2021-12-10

**Authors:** Truong Xuan Dai, Hoang Nghia Son, Ho Nguyen Quynh Chi, Hoang Nghia Quang Huy, Nguyen Thai Minh, Nguyen Thi Thuy Tram, Nguyen Thi Thuong Huyen, To Minh Quan, Doan Chinh Chung, Truong Hai Nhung, Tran Thi Minh, Tran Hong Diem, Nguyen Thi Phuong Mai, Le Thanh Long

**Affiliations:** 1 Animal Biotechnology Department, Institute of Tropical Biology, Vietnam Academy of Science and Technology, Ho Chi Minh City 700000, Vietnam; 7truongxuandai@gmail.com (T.X.D.); hoangnghiason@yahoo.com (H.N.S.); quynhchihonguyen@gmail.com (H.N.Q.C.); hoangnghiaquanghuy@gmail.com (H.N.Q.H.); tminh0602@gmail.com (N.T.M.); ttramnt@gmail.com (N.T.T.T.); doanchinhchung@gmail.com (D.C.C.); 2 Biotechnology Department, Graduate University of Science and Technology, Vietnam Academy of Science and Technology, Ha Noi 100000, Vietnam; tomquan@hcmus.edu.vn; 3 Department of Animal Physiology, Biology Faculty, Ho Chi Minh City University of Education, Ho Chi Minh City 700000, Vietnam; huyenntth@hcmue.edu.vn; 4 Faculty of Biology and Biotechnology, University of Science, Ho Chi Minh City 700000, Vietnam; thnhung@hcmus.edu.vn; 5 Technology Department, Van Lang University, Ho Chi Minh City 700000, Vietnam; minh.tt@vlu.edu.vn; 6 Children Research Institute, UT Southwestern Medical Center, 6000 Harry Hines Blvd., Suite NL11120C, Dallas, TX 75390, USA; DiemH.Tran@utsouthwestern.edu; 7 Tay Nguyen Institute for Scientific Research, Vietnam Academy of Science and Technology, Da Lat City 66000, Vietnam; phuongmaipvsh@yahoo.com

**Keywords:** cell cycle proteins, cell morphology, porcine granulosa cells, proliferation, simulated microgravity

## Abstract

Astronauts are always faced with serious health problems during prolonged spaceflights. Previous studies have shown that weightlessness significantly affects the physiological function of female astronauts, including a change in reproductive hormones and ovarian cells, such as granulosa and theca cells. However, the effects of microgravity on these cells have not been well characterized, especially in granulosa cells. This study aimed to investigate the effects of simulated microgravity (SMG) on the proliferation and morphology of porcine granulosa cells (pGCs). pGC proliferation from the SMG group was inhibited, demonstrated by the reduced O.D. value and cell density in the WST-1 assay and cell number counting. SMG-induced pGCs exhibited an increased ratio of cells in the G0/G1 phase and a decreased ratio of cells in the S and G2/M phase. Western blot analysis indicated a down-regulation of cyclin D1, cyclin-dependent kinase 4 (cdk4), and cyclin-dependent kinase 6 (cdk6), leading to the prevention of the G1-S transition and inducing the arrest phase. pGCs under the SMG condition showed an increase in nuclear area. This caused a reduction in nuclear shape value in pGCs under the SMG condition. SMG-induced pGCs exhibited different morphologies, including fibroblast-like shape, rhomboid shape, and pebble-like shape. These results revealed that SMG inhibited proliferation and induced morphological changes in pGCs.

## 1. Introduction

Gravity plays an important role in the evolution and development of life on Earth [1]. To date, more than 559 humans have traveled to space since 1961 [2]. Astronauts are exposed to multiple potential hazards to reproductive function from extended travel in space [3]. The previous study reported that rat spermatogenesis was decreased by long-term exposure to microgravity during low Earth orbit and simulated microgravity on Earth [4,5]. Other studies have investigated the changes of female reproduction under SMG, by demonstrating the disruption of estrous cycling and in vitro follicle development. Space flight significantly decreases the pituitary content of LH in pregnant rats [6]. The decrease in estradiol and estrus was observed in rats under an SMG condition [7]. Space flight also induces ovulation changes and a decrease in uterine estrogen receptor expression in mice [8]. The stress endured during spaceflight may be linked to detrimental effects on the ovulatory cycle, but this has yet to be well investigated. The effects of microgravity on female reproduction have been linked to increased oxidative stress which induces changes in the cell proliferation and morphology of ovarian cells [9,10,11]. Granulosa cells play an important role in female reproduction. In the follicles, the proliferation of granulosa cells is stimulated by FSH. Estrogens also stimulate granulosa cell proliferation [12]. Granulosa cells produce estrogen during the follicular phase and progesterone after ovulation [13]. After ovulation, granulosa cells are transformed into luteal cells which are essential for the implantation and early development of the zygote [14]. The changes in the function and structure of granulosa cells may trigger stress in female physiology. 

The effects of microgravity on granulosa cells have been unclear. In this study, pGCs were used for evaluating the effect of simulated microgravity on the proliferation and morphology of granulosa cells. This cell type was applied to this study because it showed numerous advantages. Firstly, pigs have many shared similarities to humans including size, physiology, anatomy, metabolic profile, and longer lifespan [15,16,17,18] and they have been developed to be the superior models of human conditions [17]. Pigs are one of the most important models in biomedical research, especially in developmental biology and reproductive biology [19]. Secondly, pGCs are widely used to study granulosa cell function [20]. The proliferation and differentiation of pGCs have been well characterized under a variety of culture conditions [21,22,23,24,25]. pGCs are also used for the establishment of the induced immortal granulosa cell line [26]. Moreover, pGCs are a common model for studying autophagy and apoptosis of granulosa cells [27,28,29]. Therefore, pGCs have shown advantageous characteristics which are suitable to study changes of granulosa cells under SMG conditions. 

## 2. Materials and Methods

### 2.1. pGC Isolation and Culture

Porcine ovaries were collected from the slaughterhouse and transported to the laboratory within 2 h. Single follicles were collected by a dissection method under a stereomicroscope (Meiji, Saitama, Japan) [30]. The follicles were washed in PBS (phosphate-buffered saline). The fresh pGCs were aspirated from single follicles and the oocyte-cumulus complexes were removed. The pGCs were cultured in DMEM/Ham’s F-12 (DMEM-12-A, Capricorn Scientific, Ebsdorfergrund, Germany), with 15% FBS (FBS-HI-22B, Capricorn Scientific, Ebsdorfergrund, Germany), and 1% Pen/Strep (PS-B, Capricorn Scientific, Ebsdorfergrund, Germany). 

### 2.2. Microgravity Simulation

The cell culture plates and flasks were carefully filled with culture medium (ensuring no bubbles formed to avoid fluid shearing) [31]. pGCs were induced in SMG for 72 h by Gravity Controller Gravite^®^ (AS ONE INTERNATIONAL, INC., Santa Clara, CA, USA). The Gravite has 4 programs for microgravity simulation including mode A (×4 rpm), mode B (×3 rpm), mode C (×2 rpm), and mode D (×1 rpm), in which mode C was recommended for cell culture. Thus, this study applied the mode C operation of the Gravite for microgravity simulation in pGCs. The Gravite^®^ operation was performed in a CO_2_ incubator (MCO-18AIC, Sanyo Electric Co., Osaka, Japan) (Supplementary Video S1). The cell plates of the SMG group were placed in Gravite on the upper tray in the CO_2_ incubator, while the cell plates of the control group (1G treatment) were placed on the lower tray of the same CO_2_ incubator. The pGCs from all groups were cultured at 37 °C, 5% CO_2_.

### 2.3. WST-1 Assay

The WST-1 assay was applied to evaluate pGC proliferation. pGCs were seeded in 96-well plates at a density of 1 × 10^3^ cells/well and induced in SMG for 72 h. After 72 h, the medium was removed, then 100 µL fresh cell culture medium (DMEM/Ham’s F-12 with 15% FBS and 1% Pen/Strep) and 10 µL WST-1 solution (11644807001, Roche, Basel, Switzerland) were added to each well. pGCs were incubated at 37 °C, 5% CO_2_ for 3.5 h. The optical density 450 (O.D. 450) was measured by the GloMax^®^ Explorer Multimode Microplate Reader (Promega, Fitchburg, WI, USA).

### 2.4. Cell Density Determination

pGCs were seeded in 96-well plates at a density of 1 × 10^3^ cells/well. pGCs were cultured in DMEM/Ham’s F-12 with 15% FBS and 1% Pen/Strep and induced in SMG for 72 h. After 72 h, pGCs were fixed with 4% paraformaldehyde in PBS (Nacalai Tesque, Kyoto, Japan) for 30 min and permeabilized with 0.1% Triton X-100 in PBS (Merck, Darmstadt, Germany) for 30 min. The pGCs nuclei were stained with Hoechst 33342 (14533, Sigma-Aldrich, Munich, Germany) for 30 min. Cell washing with PBS was performed three times in 5 min for each step. Cell number was determined by nuclei counting with the cell cycle app. of the Cytell microscope (GE Healthcare, Chicago, IL, USA).

### 2.5. Flow Cytometry

pGCs were seeded in T-25 flasks at a density of 1 × 10^5^ cells/flask. pGCs were cultured in DMEM/Ham’s F-12 with 15% FBS and 1% Pen/Strep and induced in SMG for 72 h. To estimate cell cycle progression, pGCs were fixed with 4% paraformaldehyde (09154-85, Nacalai Tesque, Kyoto, Japan) for 15 min and resuspended in cold PBS. pGCs were stained with 5 µL PI (5166211E, BD Biosciences, San Jose, CA, USA). Cell cycle analysis was performed by measuring the cellular DNA content using a flow cytometer BD Accuri C6 Plus (BD Biosciences, San Jose, CA, USA). To evaluate the viability and apoptosis of pGCs, the flow cytometry analysis was performed by FITC Annexin V Apoptosis Detection Kit I (556547, BD Biosciences, San Jose, CA, USA) by a BD Accuri C6 Plus cytometer (BD Biosciences, San Jose, CA, USA).

### 2.6. Western Blot

pGCs were seeded in T-25 flasks at a density of 1 × 10^5^ cells/flask. pGCs were cultured in DMEM/Ham’s F-12 with 15% FBS and 1% Pen/Strep and induced in SMG for 72 h. pGC lysate was prepared with an Optiblot LDS Sample Buffer (ab119196, Abcam, Cambridge, MA, USA) and was loaded into the Precast Gel SDS-PAGE 4–12% (ab139596, Abcam, Cambridge, MA, USA). The electrophoresis was performed in an Optiblot SDS Run Buffer (ab119197, Abcam, Cambridge, MA, USA) for 2 h at 50 V. The protein was transferred to a methanol-treated PVDF membrane (ab133411, Abcam, Cambridge, MA, USA). The membrane was treated with blocking buffer (ab126587, Abcam, Cambridge, MA, USA). The membrane was incubated with primary antibodies overnight at 4 °C. Anti-Cdk4 (ab137675, Abcam, Cambridge, MA, USA), anti-Cdk6 (ab124821, Abcam, Cambridge, MA, USA), and anti-Cyclin D1 (ab40754, Abcam, Cambridge, MA, USA) were used at a 1:5000 dilution. Anti α-tubulin (ab52866, Abcam, Cambridge, MA, USA) was used at a 1:10,000 dilution. The membrane was washed three times with 1X TBST. The membrane was incubated with goat anti-rabbit IgG (HRP) (ab6721, Abcam, Cambridge, MA, USA) at room temperature for 1 h, then was washed three times with 1X TBST. The blots were visualized using a ECL Western Blotting Substrate Kit (ab65623, Abcam, Cambridge, MA, USA). Imaging was carried out with an X-ray film in a darkroom.

### 2.7. Microfilament Staining

Four percent paraformaldehyde in PBS (Nacalai Tesque, Kyoto, Japan) was applied to fix the pGCs at room temperature for 30 min, then cells were permeabilized with 0.1% Triton X-100 in PBS (Merck, Darmstadt, Germany) at room temperature for 30 min. The pGCs were washed with PBS three times in 10 min between each step. Phalloidin CruzFluor™ 488 conjugate (Santa Cruz Biotechnology, Santa Cruz, CA, USA) was used for actin filament staining in 1 h. The pGC nucleus was counterstained with Hoechst 33342 (14533, Sigma-Aldrich, Munich, Germany) for 30 min and then washed with PBS three times in 5 min for each step. The pGCs were observed under a Cytell microscope (GE Healthcare, Chicago, IL, USA) for changes in nuclear and cell morphology.

### 2.8. Statistical Analysis

Statistical analyses were performed with Sigma Plot 11.0 (Systat Software Inc., San Jose, CA, USA. The data were analyzed for statistical significance with a one-way ANOVA, where *p* < 0.05 was considered statistically significant.

## 3. Results

### 3.1. SMG Inhibited the Proliferation of pGCs

**Figure 1:**
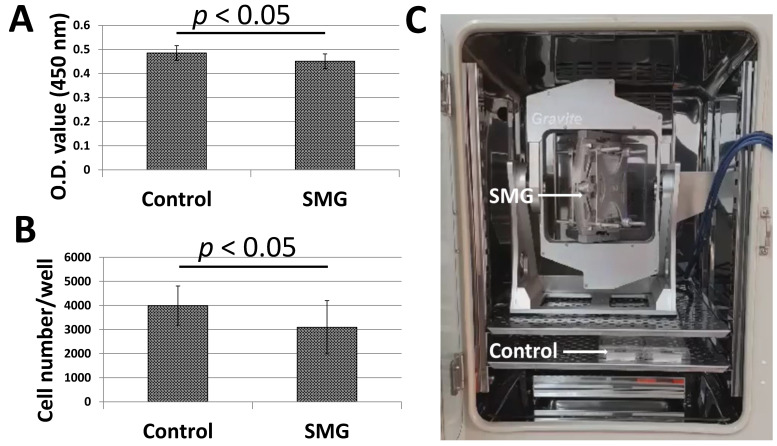
The proliferation of pGCs from the control and SMG groups. (**A**) pGC proliferation assessed by a WST-1 assay; (**B**) pGC density assessed by the cell cycle app. of a Cytell microscope; (**C**) Gravite operation in a CO_2_ incubator.

Figure 1A,B illustrated the proliferation of pGCs under SMG induced by the Gravite simulator (Figure 1C). pGCs of the SMG group showed lower expansion than the control group. The WST-1 assay was applied to estimate pGC proliferation. The absorbance value of the pGCs from the SMG group was 0.45 ± 0.03, which was lower than cells in the control group (0.49 ± 0.03, *p* < 0.05) (Figure 1A and Table S1). The proliferation of pGCs was also estimated by the determination of the cell number/well. The number of pGCs from the control group was 3995 ± 815 cells/well, which was higher than the SMG group (3095 ± 1108 cells/well, *p* < 0.05) (Figure 1B and Table S2). This result indicated that the proliferation of pGCs was reduced under the SMG condition. 

**Figure 2:**
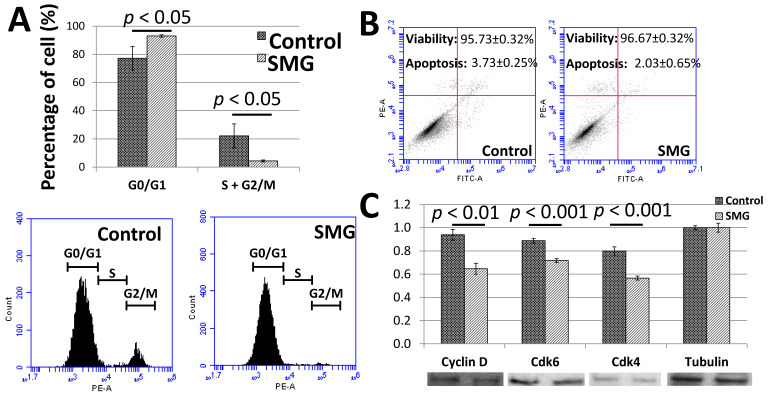
Cell cycle progression of pGCs. (**A**) Cell cycle analysis of pGCs evaluated by flow cytometry; (**B**) the viability of pGCs was assessed by flow cytometry; (**C**) the expression of major cell cycle-related proteins was estimated by Western blot.

To clarify the low proliferation of pGCs under the SMG condition, the cell cycle progression was assessed to evaluate the ability of cells to divide. The cell cycle progression of pGCs was evaluated by flow cytometry. As seen in Figure 2A and Table S3, the G0/G1 ratio of pGCs from the SMG group was 93.07 ± 0.86%, which was higher than the control group (77.25 ± 8.41%). Oppositely, the total ratio of S and G2/M phases of the pGCs from the SMG group was lower than the control group (4.37 ± 0.70% vs. 22.15 ± 8.56%). This result revealed that SMG can induce an increase in the ratio of pGCs in the G0/G1 phase which lead pGCs into the cell cycle arrest phase. 

The flow cytometry analysis also indicated a slight increase in the viability ratio of pGCs from the SMG group. The viability ratios of pGCs from the control and SMG group were 95.73 ± 0.32% and 96.67 ± 0.32%, respectively. The apoptosis ratio of pGCs from the SMG group was lower than the control group (2.03 ± 0.65% vs. 3.73 ± 0.25%, respectively) (Figure 2B). These results suggested that SMG could enhance the viability of pGCs. 

The present study also evaluated the expression of major cell cycle-related proteins. Western blot analysis showed that the expression of cyclin D1 was reduced in pGCs under the SMG condition. The down-regulation of cdk4 and cdk6 was also observed in SMG-treated pGCs (Figure 2C). This resulted in the inhibition of the G1/S phase transition and increasing the G0/G1 ratio of pGCs under the SMG condition.

### 3.2. SMG Induced Morphological Changes in pGCs

**Figure 3:**
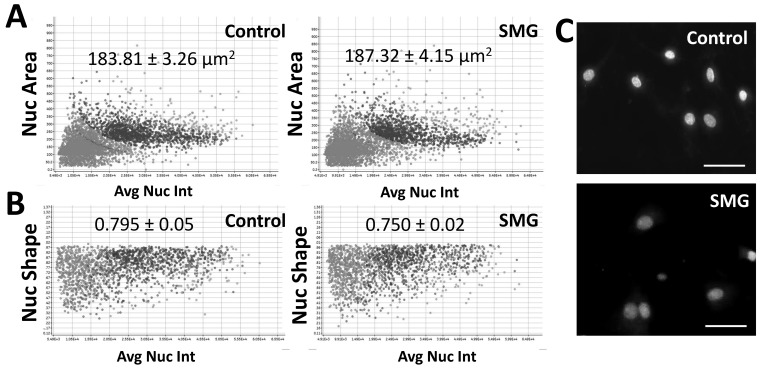
Nuclear morphology analysis of pGCs. (**A**) The distribution of the nuclear area in relation to nuclear intensity; (**B**) the distribution of the nuclear shape value in relation to nuclear intensity; (**C**) nuclei were counterstained with H33342. Scale bar = 25 µm.

In this study, we found that SMG induced morphological changes in pGCs. The changes in the nuclear morphology of pGCs are displayed in Figure 3. pGCs under the SMG condition exhibited higher nuclear area than the control group (187.32 ± 4.15 µm^2^ vs. 183.81 ± 3.26 µm^2^, respectively) (*p* < 0.05) (Figure 3A and Table S4). This result suggested that SMG induced an increase in the nuclear area of pGCs. 

The present work also evaluated the nuclear shape value (1.0 = circle, <1.0 = non-circular) to estimate the nuclear morphology of pGCs. As shown in Figure 3B and Table S5, the nuclear shape value of pGCs in the control group (0.795 ± 0.05) was higher than the SMG group (0.750 ± 0.02) (*p* < 0.01). This indicates the deformation of pGC nuclei in SMG conditions over the 3-day culture.

Another parameter to assess nuclear morphology was the average nuclear intensity which was generated by the cell cycle app of a Cytell Microscope (Table S6). The average nuclear intensity of pGCs from the SMG group was 987 ± 214, which was lower than the control group (1404 ± 441) (*p* < 0.01). Figure 3C illustrates that the pGC nuclear from the control group showed a higher intensity than pGCs under the SMG condition.

**Figure 4:**
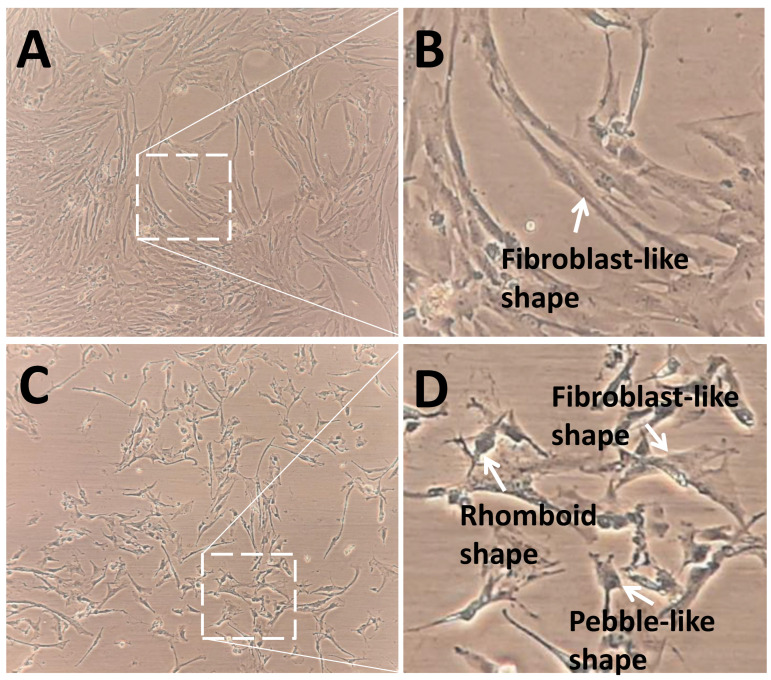
Morphology of pGCs. (**A**,**B**) pGCs from the control group showed the fibroblast-like shape; (**C**,**D**) pGCs from the SMG group exhibited various morphology, including fibroblast-like shape, rhomboid shape, and pebble-like shape. Original magnification ×100 (**A**,**C**).

**Figure 5:**
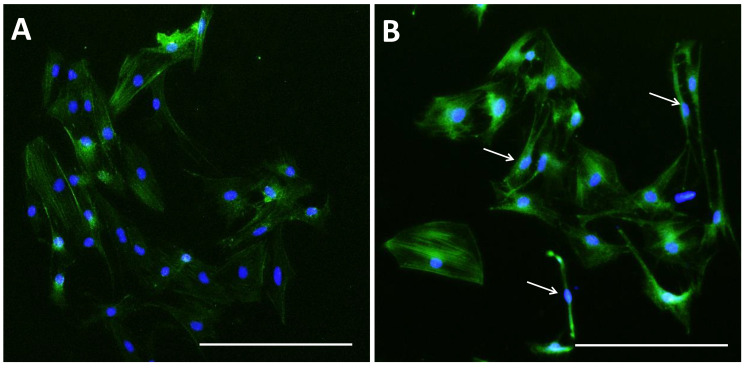
Microfilament distribution in pGCs. pGCs from the control group showed the parallel distribution of microfilament bundles (**A**); while the distribution of microfilament bundles varies with the morphology of pGCs under SMG (white arrows indicate microfilament distribution in pGCs with different shapes) (**B**). Scale bar = 223.64 µm.

The control group showed a higher density and expansion of pGCs than the SMG group (Figure 4). pGCs from the control group showed a uniform morphology with a fibroblast-like appearance (Figure 4A,B). However, pGCs from the SMG group showed different types of morphology. The fibroblast-like shape was also observed in pGCs under SMG. In addition, the rhomboid-shaped and pebble-like cells were detected in this group (Figure 4C,D). This result revealed that the SMG condition induced morphological changes in pGCs. Figure 5A demonstrates the parallel distribution of microfilament bundles in pGCs from the control group. The distribution of microfilament bundles varies with the morphology of pGCs under SMG (Figure 5B).

## 4. Discussion

The previous study, which demonstrated simulated microgravity using a rotary culture system, showed a decreased proliferation of mouse granulosa cells [32]. In the present work, we used a 3D clinostat model to generate an SMG condition. The results of this study also found that SMG inhibits pGC proliferation. This result is consistent with the above studies. Moreover, the analysis of cell cycle progression and the expression of major cell-cycle related proteins were shown to be associated with a reduction of proliferation of pGCs.

Cdk 4 and cdk6 are essential in mammalian cell proliferation, where they contribute to driving the progression of cells into the S phase of the cell-division cycle [33]. The transition from the G1 to S phase was also modulated by D-type cyclins which form complexes with cdk4 and cdk6 [34]. This investigation found that the expression of cdk4, cdk6, and cyclin D1 were reduced, resulting in the block of the G1/S transition and leading SMG-induced pGCs to the arrest of the cell cycle progression. 

SMG has been reported to show different effects on the viability and apoptosis of many cell types. An increase in the viability of cardiac progenitors was observed in cardiac progenitors under SMG [35]. Deng and colleagues demonstrated that SMG could induce apoptosis and inhibit the viability of U251 cells [36]. However, other studies demonstrated that SMG has no effects on the viability of the SAOS-2 cell line [37], adipose-derived stem cells [38], and human bone marrow mesenchymal stem cells [39]. In this study, pGCs under the SMG condition not only remained unchanged in apoptosis, but also exhibited a slight increase in viability for 72 h. This revealed that the attenuated proliferation of pGCs is not due to cell death but is caused by the down-regulation of cell cycle-related proteins and the transition to the arrest phase. 

In this study, we found that SMG induced morphological changes in pGCs. The increase in nuclear intensity is a result of chromatin condensation, which is required for cell division during mitosis [40]. The present investigation showed the decreased nuclear intensity in pGCs, suggesting that the cell division was attenuated under the SMG condition. The previous study showed that the nuclear spreading area increased under long-term SMG treatment [41]. In this work, the increase in nuclear area was observed in pGCs under the SMG condition. The change in nuclear area triggered the changes in nuclear shape, by contributing to the reduction of nuclear shape value. Thus, the deformation of nuclear was stronger in pGCs under the SMG condition. 

The nuclear shape showed a close relationship to the cytoskeleton which modulates the nuclear shape and position [41]. Microfilaments are an important component of the cytoskeleton. Actin polymerization affects the nucleus by pulling on it. Therefore, the change in the organization of microfilament bundles alters the nuclear shape. The previous study reported that MCF7 cells grow as a normal 2D monolayer under static 1g-conditions, while SMG causes the appearance of floating-clump cells and adherent cells [42]. Human adult retinal epithelium cells grow in adherent monolayers during exposure to static 1g-conditions. In contrast, these cells formed small compact round shapes under an SMG condition for 5 and 10 days on the RPM [43]. Moreover, the real microgravity generates changes in cell geometry and rapid cytoskeletal re-organization in primary human macrophages [10] These studies revealed that cell morphology can be changed under SMG conditions. In the present work, SMG induced the alteration in pGC morphology, demonstrated by the different shapes, such as fibroblast-like shape, rhomboid shape, and pebble-like shape [44]. Responding to these morphological changes, pGCs showed the reorganization of microfilament bundles which resulted in the remodeling nuclear shape. 

SMG exposure for 2 or 4 days causes the disruption of mouse ovarian secondary follicle development. The authors showed that SMG induces a decrease in the number of healthy secondary follicles and the absent proliferation of granulosa cells in secondary follicles [32]. Another study indicates that simulated weightlessness decreases follicle number, promotes follicular atresia, and inhibits the proliferation of granulosa cells. In addition, simulated weightlessness affects levels of hormone secretion and significantly reduces reproductive capacity [45,46]. Additionally, SMG caused a decrease in follicle survival and induced the down-regulation of proliferating cell nuclear antigen and growth differentiation factor 9, indicators for the development of granulosa cells and oocytes [47]. In the in vitro culture condition, outgrowing granulosa cells differentiated into fibroblast-like cells [44]. The present work found that SMG attenuated the proliferation of pGCs by diminishing the expression of cell cycle-related proteins which enhanced the transition to arrest phase. Furthermore, the cells with rhomboid shape and pebble-like shape appeared in pGC populations under SMG, suggesting that SMG reduced fibroblast-like cells and could prevent the outgrowing granulosa cells from differentiation. 

## 5. Conclusions

This study revealed that SMG inhibited the proliferation of pGCs by diminishing the expression of cell cycle-related proteins. Additionally, SMG induced morphological changes in pGCs. Further study should be conducted to estimate the effect of SMG conditions on functional changes in granulosa cells, specifically the production of hormones, as well as interactions between granulosa cells and oocytes to clarify the roles of granulosa cells during oocyte development.

## Data Availability

All data generated or analyzed during this study are included in this article.

## Supplementary Materials

The following are available online at https://www.mdpi.com/article/10.3390/cimb43030155/s1. Video S1: Gravite operation. Table S1: O.D. value; Table S2: Cell Counting; Table S3: Cell cycle progression; Table S4: Nuclear area; Table S5: Nuclear shape value; Table S6: Nuclear intensity measurement.
